# Personalized Hydration Strategy Attenuates the Rise in Heart Rate and in Skin Temperature Without Altering Cycling Capacity in the Heat

**DOI:** 10.3389/fnut.2018.00022

**Published:** 2018-04-12

**Authors:** Denise de Melo-Marins, Ana Angélica Souza-Silva, Gabriel Lucas Leite da Silva-Santos, Francisco de Assis Freire-Júnior, Jason Kai Wei Lee, Orlando Laitano

**Affiliations:** ^1^College of Physical Education, Federal University of Vale do São Francisco (UNIVASF), Petrolina, Brazil; ^2^Defence Medical and Environmental Research Institute, DSO National Laboratories, Singapore, Singapore; ^3^Yong Loo Lin School of Medicine, National University of Singapore, Singapore, Singapore

**Keywords:** dehydration, thermoregulation, performance, sports, nutrition

## Abstract

The optimal hydration plan [i.e., drink to thirst, *ad libitum* (ADL), or personalized plan] to be adopted during exercise in recreational athletes has recently been a matter of debate and, due to conflicting results, consensus does not exist. In the present investigation, we tested whether a personalized hydration strategy based on sweat rate would affect cardiovascular and thermoregulatory responses and exercise capacity in the heat. Eleven recreational male cyclists underwent two familiarization cycling sessions in the heat (34°C, 40% RH) where sweat rate was also determined. A fan was used to enhance sweat evaporation. Participants then performed three randomized time-to-exhaustion (TTE) trials in the heat with different hydration strategies: personalized volume (PVO), where water was consumed, based on individual sweat rate, every 10 min; ADL, where free access to water was allowed; and a control (CON) trial with no fluids. Blood osmolality and urine-specific gravity were measured before each trial. Heart rate (HR), rectal, and skin temperatures were monitored throughout trials. Time to exhaustion at 70% of maximal workload was used to define exercise capacity in the heat, which was similar in all trials (*p* = 0.801). Body mass decreased after ADL (*p* = 0.008) and CON (*p* < 0.001) and was maintained in PVO trials (*p* = 0.171). Participants consumed 0 ml in CON, 166 ± 167 ml in ADL, and 1,080 ± 166 ml in PVO trials. The increase in mean body temperature was similar among trials despite a lower increase in skin temperature during PVO trial in comparison with CON (2.1 ± 0.6 vs. 2.9 ± 0.5°C, *p* = 0.0038). HR was lower toward the end of TTE in PVO (162 ± 8 bpm) in comparison with ADL (168 ± 12 bpm) and CON (167 ± 10 bpm), *p* < 0.001. In conclusion, a personalized hydration strategy can reduce HR during a moderate to high intensity exercise session in the heat and halt the increase in skin temperature. Despite these advantages, cycling capacity in the heat remained unchanged.

## Introduction

Attenuating fluid loss during exercise in the heat is believed to be an important requirement to maintain performance. The optimal hydration strategy to be prescribed remains a matter of debate (1–4). Research demonstrate that drinking *ad libitum* (ADL) (whenever or in whatever volume desired) and drinking to thirst (relying solely on one’s personal sensation of thirst as the only stimulus or guide drinking) have similar outcomes during exercise (5, 6). A classic paradigm between these two strategies is that blood osmolality is already elevated when thirst mechanisms are activated, which might already be enough to have detrimental impact on endurance performance (7, 8). Personalizing a hydration strategy based on individual’s sweat rate appears as an alternative to the ADL and drink to thirst approaches (1, 3), but its influence on physiological and thermoregulatory parameters requires further investigation.

Among the potential advantages of personalizing the hydration strategy are the avoidance of under- and overdrinking, which can hamper performance and health (7). To personalize a hydration strategy requires a pre-assessment of sweat rate, which varies according to exercise intensity and environmental condition and may require athletes and sports nutritionists to periodically monitor changes in body mass in different instances and to make adjustments in the drinking habits accordingly. Likewise, from a performance perspective during races, there is the concern for time lost if one needs to drink more during a critical part of a race/competition. Therefore, having a planned drinking strategy could be beneficial for the race tactic. Despite all these aspects, whether a personalized hydration regimen can be beneficial from a physiological perspective and can translate into positive performance outcomes remains unknown. Research in this field have been inconclusive so far (2, 4). For instance, while one study demonstrated improvements in 16 km running performance with ADL drinking regimen in comparison with a personalized volume (PVO) strategy (4), another study did not find differences between these two strategies (2). Conversely, a recent investigation showed improvements in 5 km simulated uphill cycling performance with a personalized drinking strategy in comparison with ADL (3). The conflicting outcomes from these studies could be due to several factors such as pre-hydration status (i.e., eu vs. hypohydrated), participants’ fitness level (i.e., elite vs. recreational), types of drink consumed (i.e., water vs. sports drink), and exercise mode (i.e., running vs. cycling).

Recently, it has been suggested that the success of a given hydration strategy during exercise may also depend on the context of the exercise/sport (duration, intensity, and environment), the characteristics of the individual (fitness and acclimatization status), and the goals of the individual exercising, training, or competing (1). The aim of the present investigation was to test whether personalizing a volume of water based on sweat rate would have beneficial effects on physiological responses and exercise capacity during a moderate duration, high intensity cycling to volitional exhaustion in the heat.

## Materials and Methods

### Participants and Ethical Aspects

Eleven male recreational cyclists (age = 30 ± 7 years; height = 1.77 ± 0.01 m; body mass = 74.7 ± 10.6 kg; body fat = 11.7 ± 0.5%) were recruited. They had at least 3 years of experience with road or mountain bike cycling. Participants visited the laboratory on five separate occasions. They were asked to record their 24 h food intake and then to repeat the same intake before each visit. The study protocol was approved by the University Research Ethics Committee under the number 0015/250614. All participants gave written informed consent after being informed about the risks involved with the study. All procedures conformed to the code of Ethics of the Medical Association (Declaration of Helsinki).

### Initial Visit

During the first visit to the laboratory, participants were asked about their normal hydration regimen during exercise to identify any habit that could help with the elaboration of the experimental hydration plan. Thereafter, semi-nude body mass (Marte scale LS200, Brazil) and height were recorded before we performed skinfold assessment (Scientific Skinfold Caliper, Cescorf, Brazil) of the body composition to determine participants’ anthropometrical characteristics. Nine skinfolds (biceps, triceps, subscapular, chest, axillar, abdomen, supra iliac, thigh, and calf) were assessed in triplicate by an experienced lab member and percentage body fat was estimated by using the equation proposed by Jackson and Pollock (9). Thereafter, participants undertook a progressive incremental cycling test until exhaustion (CEFISE, model Biotec 200, Brazil). The protocol consisted of an initial workload of 30 W with increments of 30 W/min until exhaustion. The cadence was kept between 60 and 70 rpm, and heart rate (HR) was continuously monitored by a telemetry band (Polar, model S610, Polar Electro, Finland). The test was performed in a warm room with temperature of 34°C and relative humidity of 40% to promote habituation to mechanical, stationary cycling in the heat. Another objective of this visit was to enhance cardiovascular stability and to reduce the interindividual variability in the measurements.

### Familiarization and Sweat Rate Assessment

Participants performed two familiarization cycling sessions at 70% of the maximal workload achieved in the previous incremental test (~85% of HR_max_) for 45 min to minimize learning effects throughout the study and to enhance their heat acclimatization profile. The two sessions were interspersed by at least 2 and at most 4 days to allow appropriate recovery. A fan was positioned at 120 cm of the front of the ergometer with a wind speed of 2.5 m/s to help increase sweat rate due to enhanced convective gradient (10). The session was performed in the heat to mimic the subsequent experimental trials (34°C and 40% relative humidity). Participants’ sweat rate was determined during the second familiarization trial based on the change in body mass adjusted by time. This sweat rate was taken into consideration for the calculation of the PVO trial. Percentage (%) dehydration was calculated by multiplying post-exercise body mass by 100 and then dividing it by pre-exercise body mass and subtracting it from 100. Sweat loss was calculated by subtracting the initial from the final body mass in kilograms and then converting to liters, assuming 1 kg corresponds to 1 l. The volume of fluid consumed was added, and urine production was accounted in the equation.

### Experimental Hydration Plan

We elaborated a personalized experimental hydration regimen with water based on sweat rate previously assessed in the familiarization session. As participants were not accustomed to large fluid intakes, our goal was to achieve a volume of fluid to offset 80% of the total sweat loss assessed in the previous familiarization session. During the PVO trial, the calculated volume was then administered at a rate of 3.5 ml of water/kg body mass every 10 min.

### Experimental Trials

Five days after the last familiarization session, participants performed three experimental trials in the heat. The trials were randomized and interspersed by at least 1 week to avoid the effects of learning and carry over effects of residual fatigue from the previous exercise session, respectively. The exercise intensity and environmental conditions were identical to those previously described in the familiarization sessions. Participants were instructed to self-insert a rectal probe (Physitemp Instruments, New Jersey, NJ, USA) 10 cm past the sphincter to monitor core temperature during each session. Skin temperature sensors (iButton, Maxim Integrated, San Jose, CA, USA) were placed on the chest, upper back, lower back, forearm, thigh, and calf. The sensors were maintained adhered to the skin, despite the presence of profuse sweating, by using a special tape permeable to air and moisture (Fixomull Stretch Tape). Skin temperature was calculated by using the equation proposed by Ramanathan (11). Mean body temperature (MBT) was determined by Hardy and Dubois equation (12): MBT = 0.8 × *T*_rectal_ + 0.2 × *T*_skin_, where *T*_rectal_ = rectal temperature and *T*_skin_ = skin temperature. In the PVO trial, participants were prescribed 3.5 ml/kg every 10 min at minutes 0, 10, 20, and 30. For the ADL trial, bottles containing 800 ml of water were available for participants to drink upon request. Drink temperature for both trials was kept at ~10.5°C to promote consumption (13). In the control trial (CON), participants did not have access to drinks. Cycling time to exhaustion was recorded and used as an indicator of exercise capacity. No verbal encouragement was provided in any trial throughout the study. The test was halted when participants were unable to maintain the cadence. Before exercise, 5 ml blood samples were collected from an antecubital vein while participants were comfortably seated in a semi-recumbent position. Immediately after exercise, participants left the cycle ergometer and sat comfortably again for another 5 ml blood sample. These samples were centrifuged at 2,000 rpm for 10 min used to determine serum osmolality (Microprocessed Osmometer, PZL-1000, Brazil).

### Statistical Analysis

A sample size calculation was initially performed with G*Power software taking into consideration previously observed effects size and the requirements of a significance level of *p* < 0.05 from studies determining exercise performance (4, 5). Normality of data distribution was assessed by Shapiro–Wilk test. Repeated measures analysis of variance with Tukey’s *post hoc* was performed to compare means among trials. Two-way analysis of variance was employed to compare delta values in temperature and HR. Paired *t*-test was performed to compare the fluid consumption and body mass loss among trials. Data are reported as mean ± SD followed by the degrees of freedom unless otherwise stated. For the purpose of hypothesis testing, the 95% level of confidence was predetermined as the minimum criterion to denote a statistical difference (*p* < 0.05). All data analyses were undertaken using SAS JMP PRO 13 and GraphPad Prism software.

## Results

**Figure 1:**
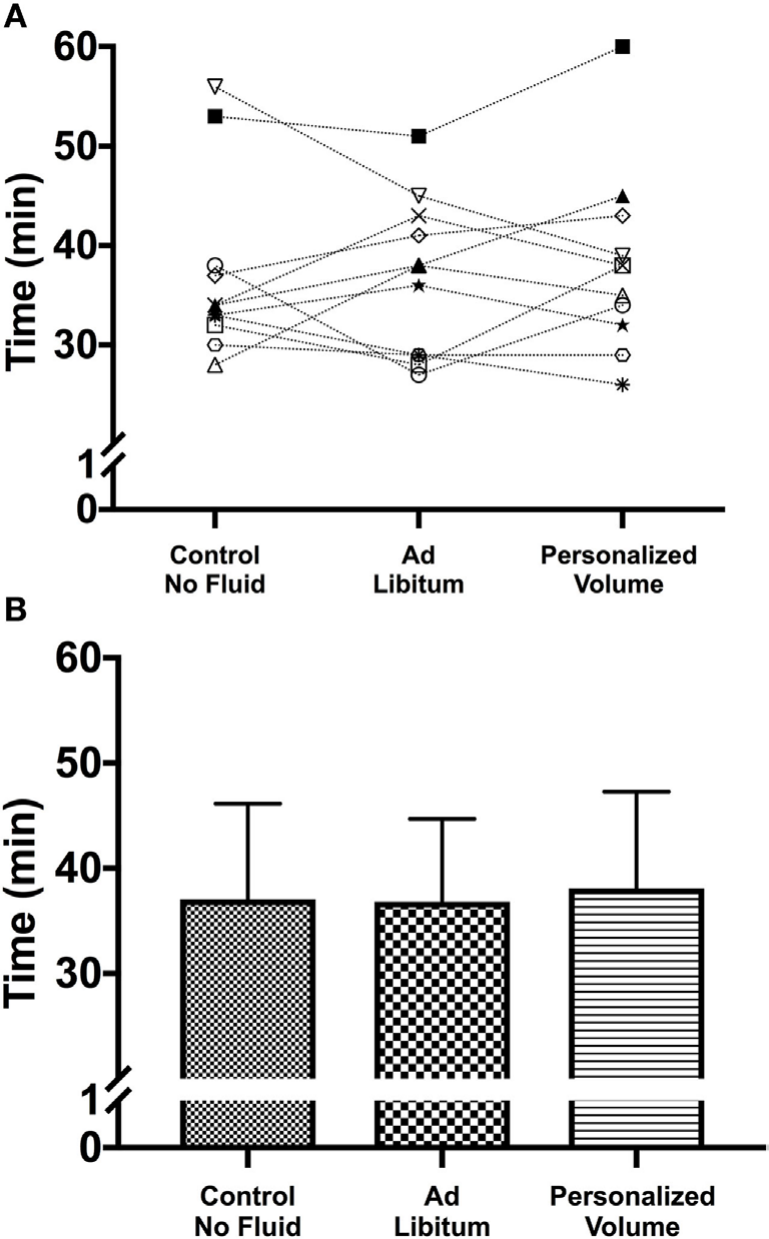
**(A)** Individual performance in the time-to-exhaustion trial in the heat for the three different hydration strategies. Despite the absence of significant differences among hydration strategies it is possible to identify participants whose performance was positively or negatively affected by the personalization of the hydration strategy. **(B)** Mean and SD for each of the experimental trials.

**Figure 2:**
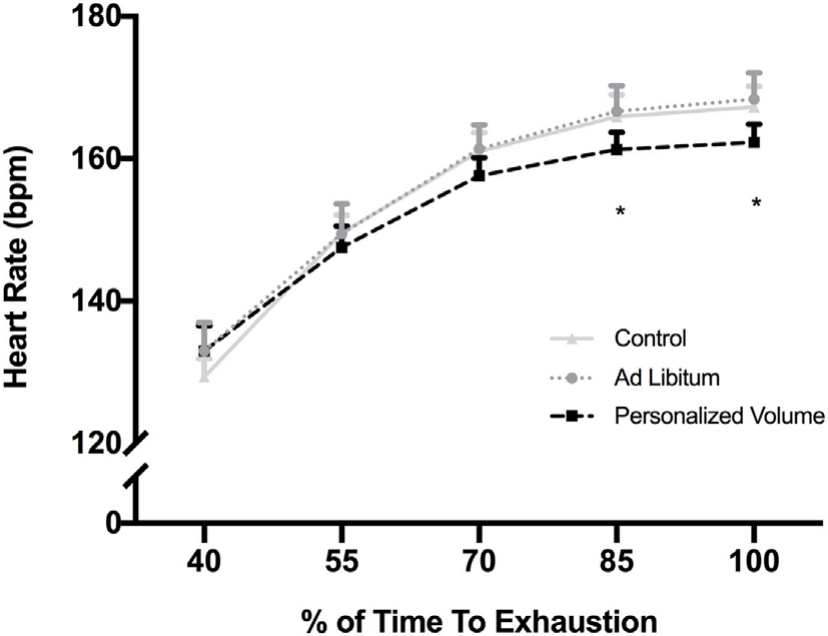
Heart rate response during exercise in the heat for each of the experimental trials. *p < 0.05 in comparison with control and *Ad Libitum* trials.

During the incremental test, participants achieved a maximal power output of 372 ± 30 W. Therefore, participants workload during the trials was on average 260 ± 30 W. As described in Table 1, participants started all trials with similar hydration status based on urine-specific gravity (USG) and blood osmolality. As observed in Figure 1, the time taken to complete all three time-to-exhaustion (TTE) trials was similar regardless of the strategy [37 ± 8 min in the ADL trial, 38 ± 9 min in the PVO trial, and 37 ± 9 min in the CON trial, *p* = 0.801, *F*(1.8, 18) = 0.196] and despite the differences in fluid ingestion and body mass. Cyclists ingested 166 ± 167 ml of fluids in the ADL trial, which was much lower than the volume consumed in the PVO trial, 1,080 ± 166 ml, *p* < 0.001, *t* = 20.06, df = 11. Since we did not provide fluids in the control trial, the fluid consumption was 0. The volume of fluid ingested impacted the hydration status during exercise. In the CON and ADL trials, we observed a reduction in body weight (CON from 75.4 ± 10.3 to 74.5 ± 10.4 kg, *p* < 0.001, *t* = 4.831, df = 11; ADL from 75.4 ± 10.4 kg to 74.6 ± 10.6, *p* = 0.008, *t* = 3.171, df = 11), whereas in the PVO trial body mass did not change (from 75.65 ± 10.18 to 75.52 ± 10.16, *p* = 0.171, *t* = 1.462, df = 11). The changes in body mass reflected the percentage of dehydration which was greater in CON and ADL in comparison with PVO [*p* = 0.007, *F*(1.8, 20.5) = 6.38] as described in Table 1. Blood osmolality did not change after the exercise trial regardless of the hydration strategy employed. Although the increase in core temperature and MBT was similar among all trials, we observed a smaller increase in skin temperature in the PVO trial in comparison with CON, *p* = 0.038, *F*(2, 15) = 4.70. Toward the end of the exercise trials, we observed a smaller increase in HR in the PVO trial (162 ± 8 bpm) in comparison with both CON (167 ± 10 bpm) and ADL (168 ± 12 bpm), *p* < 0.001, *F*(4, 120) = 229.5, as demonstrated in Figure 2.

**Table 1:** Urine-specific gravity (USG), serum osmolality, sweat rate, dehydration, and changes in temperatures at the three experimental conditions.

	Control	Ad Libitum	Personalized volume
Pre-exercise USG	1.025 ± 7.6	1.025 ± 7.2	1.026 ± 5.6
Pre-exercise serum osmolality (mOsm/kg)	296 ± 4	294 ± 3	296 ± 4
Post-exercise serum osmolality (mOsm/kg)	297 ± 7	298 ± 6	298 ± 6
Pre-exercise body mass (kg)	75.4 ± 10.3	75.4 ± 10.4	75.6 ± 10.2
Post-exercise body mass (kg)	74.5 ± 10.4	74.6 ± 10.6	75.5 ± 10.2
Sweat rate (l/h)	1.5 ± 0.4	1.4 ± 0.3	1.9 ± 0.5
Dehydration (%)	1.3 ± 0.6	1.0 ± 0.5	0.2 ± 0.4^#^
Δ Core temperature (°C)	0.6 ± 0.3	0.6 ± 0.3	0.5 ± 0.3
Δ Skin temperature (°C)	2.9 ± 0.5	2.6 ± 0.9	2.1 ± 0.9*
Δ Mean body temperature (°C)	1.0 ± 0.3	1.0 ± 0.3	0.9 ± 0.4

*p < 0.05 in comparison with control.

*^#^p < 0.05 in comparison with control and ad libitum*.

*Data are mean ± SD*.

## Discussion

The main goal of this study was to determine the impact of a personalized hydration strategy with water on cardiovascular, thermoregulatory and exercise capacity of recreational cyclists while exercising in the heat. Our main finding was that the personalized strategy resulted in lower HR and skin temperature during exercise in the heat and attenuated the degree of dehydration. Importantly, exercise capacity was similar regardless of the hydration strategy employed. These findings add relevant information to previous studies reporting conflicting results on the importance of hydration strategies on physiological outcomes involved in exercise performance in warm environments (2–4, 14).

Hydration guidelines for athletes exercising in the heat encourage consumption of sufficient amounts of fluids during exercise to limit water and salt deficits and also to avoid over drinking (15, 16). Our initial hypothesis was that personalized strategy would have a positive impact in exercise capacity in the heat when compared with ADL fluid intake. Conflicting results have recently been reported in the literature (2–4). For instance, Rollo et al. (4) investigated the effect of ADL versus PVO carbohydrate-electrolyte solution intake on 16 km running performance. Running performance was improved in the ADL trial despite the greater carbohydrate and electrolyte intake in the PVO trial. As they used carbohydrate solution it is difficult to establish a parallel with our model (e.g., hydration with water). Nevertheless, it has been suggested that runners are able to better replace fluid losses in ADL trials in comparison with cyclists (17), which might explain the improved running performance in Rollo et al. study. Conversely, Lopez et al. (2) also investigated the effects of ADL and PVO in euhydrated runners and did not find differences in the time taken to complete 20 km between the two strategies. More recently, Bardis et al. (3) studied the impact of personalized versus ADL hydration strategies on a simulated 5 km high intensity cycling trial. Their findings suggested a performance advantage in the personalized drinking trial. Despite the differences in exercise mode (cycling vs. running), intensity, and type of hydration solution, our results corroborate with Lopez et al. and suggest that performance during exercise in the heat is similar between ADL and PVO hydration strategies. In addition, endurance capacity was not degraded even when no fluid was provided in the context of our experiment.

The combination of exercise, hyperthermia, and dehydration increases cardiovascular strain (18, 19). HR is an important component of Fick equation determining oxygen consumption (VO_2_ = [stroke volume × HR] × O_2_ a-v difference) and frequently used to monitor cardiovascular response in exercise settings. Our results demonstrate an attenuated HR in the individualized, prescribed volume hydration strategy trial in comparison with ADL intake toward the end of the exercise trial. This occurred along with a lesser degree of dehydration observed in the PVO trial. The hypothesis that personalized strategy would improve endurance capacity was not confirmed in our study. Core temperature was similar regardless of the hydration strategy employed. Even though the total amount of water consumed was significantly higher in the PVO trial, both ADL and no fluid (control) trials elicited significant reductions in body mass that were still within the 2% threshold often considered to negatively influence performance (20) and to affect thermoregulatory responses (21–23). The cutoff of 2% decrease in body mass is believed to be insufficient to impair performance in acclimated humans (24), such as the cyclists in this study. By using a more intense exercise model, Bardis et al. (3) detected positive influences of a personalized hydration regimen on cycling performance. Their participants dehydrated by 1.8% in the ADL trial. Previous research also reported dehydration levels higher than 3% with no performance impairment (23, 25). Further studies are required to confirm the hypothesis that prescribing a fixed volume of fluids based on individual sweating rates would be beneficial to humans experiencing dehydration levels >2% of body mass loss. Nevertheless, our findings seem to be in alignment with a recent statement that ADL drinking would be adequate during exercises with durations ranging from 60 to 90 min; whereas planned drinking would be recommended for longer duration activities of >90 min, particularly in the heat where performance is a concern (1).

The total volume of fluid consumed during the PVO trial was fivefold higher in comparison with the ADL trial. ADL hydration strategies are usually associated with low fluid intake (4), mainly in euhydrated participants (26). Bardis et al. reported an ADL fluid consumption of ~0.7 l in their cohort (3). In our study, particularly in the ADL trial, participants did not drink enough to offset sweat loss, which might indicate euhydration. Our participants were used to exercise in the heat and not to drink large volumes of fluid. Therefore, it is possible to conjecture that psychophysiological factors could explain the drinking patterns we observed in this study. Another interesting finding of our study was the blunted increase in skin temperature when PVO was used as hydration strategy. Skin temperature is an important variable regulating heat exchange *via* sweat evaporation (27). Even though it did not reflect in performance improvements, maintaining a lower skin temperature can be advantageous during exercise in the heat to allow heat transfer from core to the skin (28). Further studies are required to explore the mechanisms by which a lower skin temperature is observed when a PVO is adopted in comparison with ADL and CON.

A post-exercise urine sample to determine urine osmolality could add an additional marker of participants’ hydration status in addition to our current measurement of blood osmolality. It is, however, well known that urine production after exercise is directly related to renal blood flow, which is known to be markedly reduced during exercise, especially in the heat where there is an increase in anti-diuretic endogenous hormones (i.e., vasopressin) (29). While a few participants provided a urine sample after exercise in the PVO trial, none was able to provide a urine sample after CON and ADL trials and therefore making the measurement of USG and osmolality not possible. Importantly, post-exercise urine osmolality has questionable sensitivity to detect hydration status (30). It is possible that the relatively short duration (~45 min) of the exercise trial coupled with the modest dehydration (~1% decrease in body mass) achieved in the ADL and CON trials could have accounted for the lack of differences among all hydration strategies in this study. Nevertheless, it is our understanding that the current results add to an already existing body of literature on the pros and cons of providing a PVO of fluids based on sweat rate to exercising humans (2, 4). It is worth to highlight that participants in our study were naturally acclimated to heat as they lived and trained in a tropical climate region. Exercise in the heat promotes long-term adaptations in sweat rate, skin blood flow, plasma volume expansion, and a greater cardiovascular stability (31, 32), which could have influenced the results herein described. Importantly, we only studied the responses of men. Whether the results of this study hold true for women requires further investigation as factors such as menstrual cycle are known to play a role in fluid balance and thermoregulation during exercise in the heat (33).

In conclusion, our study demonstrates that PVO hydration strategy was effective in reducing the cardiovascular stress and the increase in skin temperature imposed by a cycling session to exhaustion in the heat. Despite the lower cardiovascular and thermoregulatory stresses, the exercise capacity was similar regardless of the hydration strategy employed.

## Ethics Statement

This study was carried out in accordance with the recommendations of Federal University of Vale do São Francisco Research Ethics Committee. The protocol was approved by the #0015/250614. All subjects gave written informed consent in accordance with the Declaration of Helsinki.

## Author Contributions

OL and JL conceived and designed the experiment. DM-M, AS-S, FF-J, and GS-S performed the experiment. DM-M, OL, and JL analyzed and interpreted the results. DM-M, AS-S, FF-J, GS-S, JL, and OL wrote and approved the final version of the manuscript.

## Conflict of Interest Statement

The authors declare that the research was conducted in the absence of any commercial or financial relationships that could be construed as a potential conflict of interest.
